# Good Practice Statements for the treatment of nicotine dependence

**DOI:** 10.18332/tpc/167964

**Published:** 2023-07-12

**Authors:** Renata Solimini, Otto Ruokolainen, Zsuzsa Cselko, Helena Koprivnikar, Lorenzo Spizzichino, Stathis Papachristou, Adrián González-Marrón, Emilia Nunes, Dolors Carnicer-Pont, Esteve Fernandez, Anna Mar López, Elena Demosthenous, Biljana Kilibarda, Silvano Gallus, Cristina Gómez-Chacón, Ivona Keć, Maja Valentic, Hanna Ollila

**Affiliations:** 1 Istituto Superiore di Sanita, National Centre on Addiction and Doping, Rome, Italy; 2 Finnish Institute for Health and Welfare, Helsinki, Finland; 3 National Koranyi Institute of Pulmonology, Budapest, Hungary; 4 National Institute of Public Health, Slovenia; 5 Ministry of Health – Prevention Department, Rome, Italy; 6 National Public Health Organization, Athens, Greece; 7 Group of Evaluation of Health Determinants and Health Policies, Department of Basic Sciences, Universitat Internacional de Catalunya, Sant Cugat del Valles, Spain; 8 National Programme for Smoking Prevention and Tobacco Control, General-Directorate of Health, Portugal; 9 Tobacco Control Unit and WHO Collaborating Center for Tobacco Control, Catalan Institute of Oncology (ICO), L’Hospitalet de Llobregat, Spain; 10 Bellvitge Biomedical Research Institute (IDIBELL), L’Hospitalet de Llobregat, Spain; 11 Consortium for Biomedical Research in Respiratory Diseases (CIBERES), Madrid, Spain; 12 School of Medicine and Health Sciences, University of Barcelona, Barcelona, Spain; 13 Policy Department of the Cyprus National Addictions Authority, Nicosia, Cyprus; 14 Institute of Public Health of Serbia "Dr Milan Jovanović Batut", Belgrade, Serbia; 15 Istituto di Ricerche Farmacologiche Mario Negri IRCCS, Milan, Italy; 16 Ministerio de Sanidad, Madrid, Spain; 17 Croatian Institute of Public Health, Zagreb, Croatia

**Keywords:** nicotine dependence, good practice statements, nicotine cessation, new and emerging products

The current and potential future scenario regarding consumers of new and emerging tobacco and nicotine products and their sustained addiction to nicotine is a significant global public health concern. To address this issue, it is crucial to incorporate individuals addicted to these products into the existing treatments for smoking cessation, as per the Good Practice Statements (GPS) provided by the GRADE working group^1-3^.

GPS consist of actionable statements that can bring about substantial benefits or harms, but do not undergo the formal assessment of evidence quality as required by the GRADE method for formulating recommendations. While formulating GPS, supporting literature should be available, such as reports from international organizations or agencies, reviews, case reports, or clinical trials. The GRADE working group has proposed five criteria to evaluate the appropriateness of issuing GPS^3^, distinguishing them from GRADE recommendations:

1. Statement is clear and actionable;
2. Message is necessary regarding healthcare practice;
3. Implementation of the statement is likely to result in large net positive consequences;
4. Summarization of evidence would be a poor use of guideline panel’s time; and
5. The rationale connecting the indirect evidence used to support the statement is clear and explicit.

In situations where studies do not include users of various tobacco and nicotine products like electronic cigarettes, heated tobacco products, or other emerging products containing tobacco and/or nicotine with addictive potential, it may be necessary to develop GPS to address nicotine dependence. The interventions recommended in these GPS are based on existing evidence-based interventions for smoking cessation, including pharmacotherapy, counselling, and digital interventions. However, it is important to exercise caution when considering the inclusion of vulnerable groups such as children, youth, and pregnant women, and it should be done on a case-by-case basis.

For consumers of heated tobacco products, smokeless tobacco and nicotine-containing products (e.g. electronic cigarettes, nicotine pouches or any other new and emerging nicotine product with an addictive potential), the proposed GPS are the following:

GPS 1. It is reasonable to give brief advice for cessation. Brief cessation advice should consist of asking about the details of product use [type(s) of product(s), frequency of use, nicotine content, and duration of use], advising to quit, and assessing readiness to quit^4-6^.

For consumers motivated to quit:

GPS 2. It is reasonable to offer pharmacotherapy to treat nicotine withdrawal symptoms. Pharmacotherapy includes the use of nicotine replacement therapy (NRT) in its different formulations and/or combinations (slow release and rapid release), as well as varenicline. It is also possible to use bupropion, or alternatively cytisine, according to clinical considerations and patient choice^4-13^.

GPS 3. It is reasonable to offer individual or group counselling combined with pharmacotherapy. The choice of pharmacotherapy depends on clinical considerations and patient preferences^4-7,9-14^.

GPS 4. It is reasonable to consider nicotine replacement therapy (NRT) with counselling in youth or pregnant women unable to quit with counselling alone^15^.

GPS 5. It is reasonable to use a digital intervention (via applications on mobile phones, text messages, online programs)^8,14,16-20^.

The rationale behind the aforementioned GPS is primarily grounded in the understanding that effective treatments, both pharmacological and behavioral, for nicotine addiction in traditional cigarette smokers should also be applicable to individuals using new and emerging tobacco and nicotine products. Nicotine is the addictive substance present in both conventional tobacco and its derivatives or alternative products. Therefore, when attempting to cease the use of these various new or emerging products containing nicotine, it is necessary to consider medications that act on nicotinic receptors, just as it is the case with conventional tobacco cigarettes. NRT (nicotine replacement therapy) and varenicline, which specifically target α4β2 receptors (in the case of varenicline), meet these requirements and can be effective options for treatment. Additionally, the use of cytisine, a partial agonist of the α4β2 receptor (similar to varenicline), shows promise and may be a suitable choice^13,21^. There are several studies supporting the effectiveness of NRT and varenicline for the treatment of smokeless tobacco addiction, as well as case reports demonstrating their efficacy in treating electronic cigarette addiction^9,12^. These studies explicitly highlight the positive outcomes of using NRT and varenicline. On the other hand, the suggestion to use bupropion and cytisine for electronic cigarette cessation or the cessation of other nicotine-containing products is primarily based on an intuitive approach, indicating that effective treatments for smoking cessation could potentially be effective for these cases as well^5,13,14^. Therefore, the use of bupropion and cytisine as alternative pharmacological treatments is suggested, taking into consideration the individual patient’s clinical characteristics on a case-by-case basis.

In the context of adolescents and youth, they are included in certain programs, literature reviews, and studies related to smoking and vaping cessation^4,5,13,14,16-18,20^. Additionally, the Centers for Disease Control and Prevention (CDC) offer comprehensive information on the use of Electronic Nicotine Delivery Systems (ENDS) and the associated risks for children, teenagers, and young adults. The CDC also provides resources and programs aimed at preventing vaping and facilitating quitting^22^. Regarding pregnant women, the American College of Obstetricians and Gynecologists (ACOG) recommends that healthcare and obstetric care professionals inquire about all forms of tobacco or nicotine use. It further emphasizes the importance of individualizing care by offering psychosocial, behavioral, and pharmacotherapy interventions to support smoking cessation during pregnancy^15^.

In conclusion, there is a pressing need for the development of GPS in healthcare practice to address nicotine dependence in individuals using non-conventional tobacco and nicotine products. These GPS should be supported by a clear and explicit rationale that connects the available indirect evidence. However, it is essential to conduct large-scale studies involving users of these products to obtain robust evidence on the appropriate and effective treatment approaches.

Currently, it is crucial to provide treatment indications in the form of GPS for all individuals addicted to these new and emerging products who are motivated to quit. These GPS are not only sustainable but also based on treatment approaches that have already demonstrated effectiveness in smoking cessation. Implementing these GPS can have positive consequences for public health, as they offer viable solutions for nicotine addiction in this specific population.

## Data Availability

Data sharing is not applicable to this article as no new data were created.
